# Effectiveness of Frequent Point-of-Care Molecular COVID-19 Surveillance in a Rural Workplace: Nonrandomized Controlled Clinical Trial Among Miners

**DOI:** 10.2196/59845

**Published:** 2025-01-27

**Authors:** Akshay Sood, William “Cotton” Jarrell, Xin W Shore, Nestor Sosa, Alisha Parada, Nicholas Edwardson, Alexandra V Yingling, Teah Amirkabirian, Qiuying Cheng, Ivy Hurwitz, Linda S Cook, Shuguang Leng, Orrin B Myers, Douglas J Perkins

**Affiliations:** 1 Department of Internal Medicine, School of Medicine, University of New Mexico Health Sciences Center, 1 University of New Mexico MSC 10 5550, Albuquerque, NM, 87131-0001, United States, 1 5052724751, 1 5052728700; 2 Miners Colfax Medical Center, Raton, NM, United States; 3 University of New Mexico Health Sciences Center, Albuquerque, NM, United States; 4 School of Public Administration, University of New Mexico, Albuquerque, NM, United States; 5 Department of Epidemiology, University of Colorado School of Public Health, Aurora, CO, United States; 6 Center for Global Health, University of New Mexico Health Sciences Center, Albuquerque, NM, United States

**Keywords:** point-of-care, seroprevalence, SARS-CoV-2, coronavirus, COVID-19, surveillance, rural workplace, miners, infectious disease, pandemic, antigen testing, midnasal swabs, public health

## Abstract

**Background:**

Numerous studies have assessed the risk of SARS-CoV-2 exposure and infection among health care workers during the pandemic. However, far fewer studies have investigated the impact of SARS-CoV-2 on essential workers in other sectors. Moreover, guidance for maintaining a safely operating workplace in sectors outside of health care remains limited. Workplace surveillance has been recommended by the Centers for Disease Control and Prevention, but few studies have examined the feasibility or effectiveness of this approach.

**Objective:**

The objective of this study was to investigate the feasibility and effectiveness of using frequent point-of-care molecular workplace surveillance as an intervention strategy to prevent the spread of SARS-CoV-2 at essential rural workplaces (mining sites) where physical distancing, remote work, and flexible schedules are not possible.

**Methods:**

In this nonrandomized controlled clinical trial conducted from February 2021, to March 2022, 169 miners in New Mexico (intervention cohort) and 61 miners in Wyoming (control cohort) were enrolled. Investigators performed point-of-care rapid antigen testing on midnasal swabs (NSs) self-collected by intervention miners. Our first outcome was the intervention acceptance rate in the intervention cohort. Our second outcome was the rate of cumulative postbaseline seropositivity to SARS-CoV-2 nucleocapsid protein, which was analyzed in the intervention cohort and compared to the control cohort between baseline and 12 months. The diagnostic accuracy of detecting SARS-CoV-2 using rapid antigen testing on NSs was compared to laboratory-based reverse transcriptase polymerase chain reaction (RT-PCR) on nasopharyngeal swabs (NPSs) in a subset of 68 samples.

**Results:**

Our intervention had a mean acceptance rate of 96.4% (11,413/11,842). The intervention miners exhibited a lower cumulative postbaseline incident seropositivity at 12 months compared to control miners (14/97, 14% vs 17/45, 38%; *P*=.002). Analysis of SARS-CoV-2 antigen detection in self-administered NSs revealed 100% sensitivity and specificity compared to laboratory-based RT-PCR testing on NPSs.

**Conclusions:**

Our findings establish frequent point-of-care molecular workplace COVID-19 surveillance as a feasible option for keeping essential rural workplaces open and preventing SARS-CoV-2 spread. These findings extend beyond this study, providing valuable insights for designing interventions to maintain employees’ safety at other essential workplaces during an infectious disease outbreak.

## Introduction

Thus far, an estimated 55 million US workers have provided essential services during the COVID-19 pandemic [1]. While the significant occupational risk of SARS-CoV-2 infection faced by essential health care workers has been well-documented [2-5], far fewer studies have investigated SARS-CoV-2 infection rates in other essential industries [6-10]. Moreover, guidance for maintaining a safely operating workplace in sectors outside of health care remains limited. Workplace surveillance has been recommended by the Centers for Disease Control and Prevention (CDC) [11], but few studies have examined the feasibility or effectiveness of this approach [12-14]. The lack of adequate data in this regard constitutes a critical gap in the literature that needs to be addressed to help devise pandemic intervention strategies for workplaces outside of health care.

Laboratory-based nucleic acid amplification tests, such as reverse transcriptase polymerase chain reaction (RT-PCR) assays performed on nasopharyngeal swabs (NPS) collected by trained professionals, are considered the gold standard for SARS-CoV-2 diagnostic testing based on accuracy, sensitivity, and specificity. However, essential workers outside the health care sector, particularly in rural and remote minority communities, may encounter issues with access, cost, and wait time for results associated with this gold standard testing. Consequently, inconsistent or absent gold standard testing has placed rural essential workers outside of health care at an increased risk for SARS-CoV-2 infection and associated complications [15].

One strategy to overcome challenges with gold standard testing is to use self-collected nasal swabs (NS) and point-of-care qualitative SARS-CoV-2 rapid antigen testing (QAT). QAT offers several advantages, such as low cost, enhanced testing accessibility, and rapid turnaround time, making it well-suited for resource-limited settings. Moreover, the use of self-collected NSs is comparable to clinician-collected NPSs for detecting SARS-CoV-2 in controlled settings [16,17]. In this study, we evaluate the diagnostic accuracy, reliability, feasibility, and effectiveness of frequent point-of-care molecular workplace COVID-19 surveillance in a diverse population of coal miners in a remote community in New Mexico, compared to a cohort of coal miners from a rural mining site in Campbell County, Wyoming. The novelty of the research question and the involvement of a diverse, understudied, and underserved population contribute to the innovation of the study. The long-term goal of the study was to inform strategies for future pandemic mitigation efforts in mining settings and other essential rural workplaces.

## Methods

### Study Design

To assess the effectiveness of implementing point-of-care QAT for SARS-CoV-2 on self-collected NS in rural workplaces, we conducted a nonrandomized controlled clinical trial involving two cohorts of coal miners from a mining site in McKinley County, New Mexico (intervention site) and from a mining site in Campbell County, Wyoming (control site). The primary recruitment period was between February 2021 and March 2022 with additional miners entering the study after this time. Inclusion criteria were as follows: male or female miners currently employed at the above-mentioned mines, at least 18 years of age, willing and able to consent to study participation, and willing and able to comply with study procedures. Exclusion criteria were as follows: unable or unwilling to consent and less than 18 years of age.

Both intervention and control sites were surface coal mines in rural and remote mountainous locations operated by the same company with similar working conditions and policies. They shared similar engineering and administrative controls and had similar protective measures on screening for SARS-CoV-2 symptoms and fever and for using face masks on-site. Workplace policy at both sites precluded miners with COVID-19 symptoms from reporting for duty, and symptom screening was conducted by mine safety personnel at the beginning of each work shift before miners could enter the mines.

Trained miner investigators collected information using phone- and computer-based web applications developed by Ingenuity Software Labs (Albuquerque, NM), paper-based questionnaires, and body temperature measurements using a no-touch forehead infrared thermometer. The information collected included a 24-hour history of SARS-CoV-2 symptoms, any provider diagnosis of infection, potential close contact with infected individuals, vaccination status, and use of cloth face covering in public settings outside the mine. Data were transferred manually by the miner investigators to the university-based investigators, who entered the information into a secure REDCap database. All information entered was double-checked for accuracy.

### Miner Testing

#### Qualitative SARS-CoV-2 Rapid Antigen Testing

For rapid detection of the SARS-CoV-2 antigen, eligible intervention miners were provided with an NS at the mine entrance at the beginning of each alternate work shift, in a schedule which consisted of 4 days on and 4 days off, resulting in 2 NSs being collected in every 8-day cycle. NSs were self-collected by the miner subjects, with trained miner investigators supervising the process. Subsequently, the 10-minute Quidel Quickvue SARS-CoV-2 QAT (ie, intervention test; Quidel Corporation, San Diego, CA) was conducted on the collected swabs by trained miner investigators at a temporary laboratory created at the intervention mine entrance. Test results were available in about 10 minutes, roughly the time required to drive from the mine entrance to the parking lot. Based on the CDC guidelines, those who tested positive were requested to return home, isolate themselves, and contact their primary care providers [11].

#### Seropositivity Testing

Venipuncture blood samples of 8 mL were obtained from intervention and control miner subjects at the time of enrollment and at 3, 6, and 12 months. Whole blood was centrifuged at the mine site, and plasma was aspirated, aliquoted, and frozen by trained nurse technicians. Plasma samples were tested to determine the seroprevalence of COVID-19 using the immunoglobulin G antibody to the nucleocapsid protein of SARS-CoV-2, and tests were performed by a qualitative chemiluminescent immunoassay (ARUP Laboratory, Salt Lake City, UT), under the Emergency Use Authorization. Unlike the antibodies to spike protein, antibodies to nucleocapsid protein develop in response to natural infection and not due to COVID-19 vaccination.

#### Diagnostic Accuracy of NSs Versus NPSs

To determine whether self-collected NSs were comparable to NPSs collected by health care providers for detecting SARS-CoV-2, a subset of miners were requested to perform a repeat self-administered NS on both nostrils, while 2 NPSs were administered by a skilled health care provider from separate nostrils, all in one setting. All swabs were placed separately into appropriately labeled tubes with viral transport media for transportation to the laboratories. The first NPS sample was used for running the confirmatory qualitative SARS-CoV-2 RT-PCR test at TriCore Diagnostic Laboratory (Albuquerque, NM)—the gold standard—performed under Emergency Use Authorization from the Food and Drug Administration. Sensitivity, specificity, and predictive values were calculated for the intervention test compared to the gold standard test.

The NS and the second NPS were used for quantitative RT-PCR (RT-qPCR) tests. Aliquots of viral transport media were inactivated with DNA/RNA Shield (Zymo Research, CA) and stored at –80°C until bulk processing using the Quick-RNA Viral kit (Zymo Research). RT-qPCR assays were performed using the N1 and RP primer/probe sets from the CDC 2019-nCoV-01 diagnostic panel. No target controls were included in all amplification reactions. The N1 primer/probe set is designed to specifically detect and amplify a region within the nucleocapsid gene of SARS-CoV-2, while the RP primer/probe set targets a portion of the human RNAse P (RNP) gene. The RP primer/probe set is used in all clinical samples to assess specimen quality. Samples with a cycle threshold (Ct) values ≥40 were considered negative. For viral load quantification, standard curves were generated from the Ct values of N1 and RP using known concentrations of SARS-CoV-2 RNA and plasmids harboring the RNP gene fragment, respectively. Viral load per sample was determined as described by Perkins et al [18]. The Cohen κ coefficient was used to determine the intrarater agreement between the NS and NPS sample collection methodologies.

### Statistical Analysis

#### Miner Characteristics

Characteristics of miners participating at the intervention and control mines were summarized as frequencies and percentages and means and SDs. Frequencies of categorical variables for the two study arms were compared using *χ*^2^ tests and Fisher exact tests. Continuous age was compared using a Wilcoxon rank sum test.

#### Intervention Acceptance

Intervention test acceptance by miners was measured for each eligible work shift, and the outcome variable and cumulative acceptance rate were calculated over the study time frame (frequency of tests accepted/number of tests offered). Predictors influencing the outcome were examined in the intervention cohort, including self-reported educational status, racial and ethnic minority status, prior SARS-CoV-2 infection or vaccination, and evidence of previous infection (from a prior positive test). Nonparametric Wilcoxon and Kruskal-Wallis tests were used to assess the association of miner characteristics with cumulative acceptance rates, since acceptance rates had a skewed distribution. We also computed the total number of tests accepted per miner for each week of the study (total number of tests accepted in each week/number of consented miners). Weekly tests per miner were summarized graphically, and a test for trend was made assuming residual errors had a first-order autocorrelation.

#### Seropositivity Analysis

Seroprevalence patterns of miners enrolled and tested between February 23 and March 6, 2021 were compared between intervention and control miners over time intervals using *χ*^2 ^tests and time-to-first seropositivity by Kaplan-Meier analysis and log-rank tests. Kaplan-Meier analyses accounted for dropouts during follow-up after baseline. We used logistic regression to compare the following binary outcome variables: baseline (prevalence) seropositivity, postbaseline incident seropositivity, and combined cumulative seropositivity (as positive at any time during the study). Combined cumulative seropositivity analyses also included miners that were enrolled after March 6, 2021. Vaccination status and select host susceptibility and vulnerability factors were added to multivariable logistic regression models. Odds ratios (ORs) and 95% CIs were computed to describe association strength. SAS version 9.4 (SAS Institute) and SPSS version 28.0.0.0 (IBM Corp) were used for statistical analyses, and a *P* value of less than .05 was considered statistically significant.

#### Power Calculation

The study was planned to estimate nasal swab acceptance rates with precision of ±4.6% from a sample of up to 250 intervention group miners, which also would have at least 80% power for subgroup analyses. A comparison group with up to n=350 was estimated to have at least 80% power to detect group differences in baseline seropositivity and incident seropositivity. However, sample sizes recruited were lower than planned.

### Ethical Considerations

The University of New Mexico Health Sciences Center’s Human Research Protections Office and Institutional Review Board (HRPO 20‐680) approved the study. All participants provided informed consent with an ability to opt out. The data were deidentified for analysis. Participants were compensated with a $10 electronic merchandise card for each specimen and survey collected.

## Results

### Study Design: Cohort Demographics

The study included 115 intervention miners and 60 miners recruited during February and March 2021 and another 54 intervention miners and 1 control miner recruited during the rest of the study (n=169 intervention and n=61 control miners). Baseline characteristics of subjects in the two cohorts are shown in Table 1. The two cohorts were similar in age distribution, with a mean age of 44.4 (SD 11.2) and 45.2 (SD 11.9) years in the intervention and control miner groups, respectively. When comparing the distribution of comorbidities, including asthma, chronic obstructive pulmonary disease, chronic lung diseases, hypertension, diabetes, and depression, 43.8% (74/169) of intervention miners reported at least 1 comorbidity, similar to 46% (28/61) of control miners.

**Table 1.** Comparison of baseline characteristics between New Mexico–based (intervention cohort) and Wyoming-based (control cohort) participants.

Characteristic	All (n=230)	New Mexico (n=169)	Wyoming (n=61)	*P* value
Age (years), mean (SD)	44.6 (11.3)	44.4 (11.2)	45.2 (12)	.57
Age group (years), n (%)				.34
<40	93 (40.4)	73 (43.2)	20 (33)	
40‐50	57 (24.8)	41 (24.3)	16 (26)	
>50	80 (34.8)	55 (32.5)	25 (41)	
Education >12th grade, n (%)	121 (52.6)	69 (40.9)	52 (85)	<.001
Annual household income ≥$80,000, n (%)	111 (48.2)	70 (41.4)	41 (67)	.005
Race, n (%)				<.001
White	152 (66.1)	96 (56.8)	56 (92)	
American Indian or Alaska Native	29 (12.6)	27 (16)	2 (3)	
Asian	1 (0.4)	1 (0.6)	0 (0)	
Other race	34 (14.8)	31 18.3)	3 (5)	
Prefer not to answer or missing	14 (6.1)	14 (8.3)	0 (0)	
Ethnicity, n (%)				<.001
Not Hispanic, Latino, or Spanish origin	117 (50.9)	63 (37.3)	54 (88)	
Hispanic, Latino, or Spanish origin	101 (43.9)	97 (57.4)	4 (7)	
Prefer not to answer or missing	12 (5.2)	9 (5.3)	3 (5)	
Biological sex at birth, n (%)				.02
Male	202 (87.8)	154 (91.1)	48 (79)	
Female	25 (10.9)	14 (8.3)	11 (18)	
Intersex, prefer not to answer or missing	3 (1.2)	1 (0.6)	2 (3)	
COVID-19-related behavior and vaccination characteristics, n (%)				
Close contact with confirmed or suspected COVID-19 since the last shift	19 (8.3)	13 (7.7)	6 (10)	.76
Frequency of ride-sharing to and from mine site often or very often	81 (35.2)	75 (44.4)	6 (10)	<.001
Frequency of face covering outside the mine often or very often	162 (70.4)	142 (84.1)	20 (33)	<.001
COVID-19 vaccination (yes)	52 (22.6)	43 (25.4)	9 (15)	.07
Comorbidities, n (%)				
Previously tested positive for COVID-19	42 (18.3)	24 (14.2)	18 (30)	.08
Asthma, COPD^a^, and other chronic lung diseases	30 (13.1)	23 (13.6)	7 (12)	.67
Hypertension	47 (20.4)	34 (20.1)	13 (21)	.87
Diabetes	21 (9.1)	17 (10.1)	4 (7)	.41
Depression	11 (4.8)	7 (4.1)	4 (7)	.46
Household wood smoke exposure (ever), n (%)	62 (26.9)	51 (30.3)	11 (18)	.08

a COPD: chronic obstructive pulmonary disease.

A significant difference was observed between the cohorts regarding racial and ethnic composition, with 72.8% (123/169) of intervention miners being from underrepresented racial and ethnic minorities (including American Indians and Hispanics), compared to 10% (6/61) of control miners (*P*<.001). The intervention cohort consisted of more male participants than the control cohort (154/169, 91% vs 48/61, 79%; *P*=.02). Furthermore, intervention miners had a lower annual income, with 58.6% (99/169) of intervention miners earning less than $80,000 annually versus 33% (20/61) of control miners (*P*=.005) and a lower education level, with 40.9% (69/169) of intervention miners having postsecondary education versus 85% (52/61) of control miners (*P*<.001). In terms of COVID-19–related behavior and vaccination characteristics, intervention miners were more likely to ride-share to and from the mining site (75/169, 44.4% vs 6/61, 10%; *P*<.001) but were also more likely to wear face coverings outside of the mine often or very often (142/169, 84.1% vs 30/61, 33%; *P*<.001). Moreover, intervention miners had a 25.4% (43/169) baseline vaccination rate against COVID-19 versus 15% (9/61) for control miners (*P*=.07). The intervention miners may have been more likely to have been exposed to household wood smoke than control miners (51/169, 30% vs 11/61, 18%; *P*=.08).

### Intervention Acceptance

**Figure 1.**
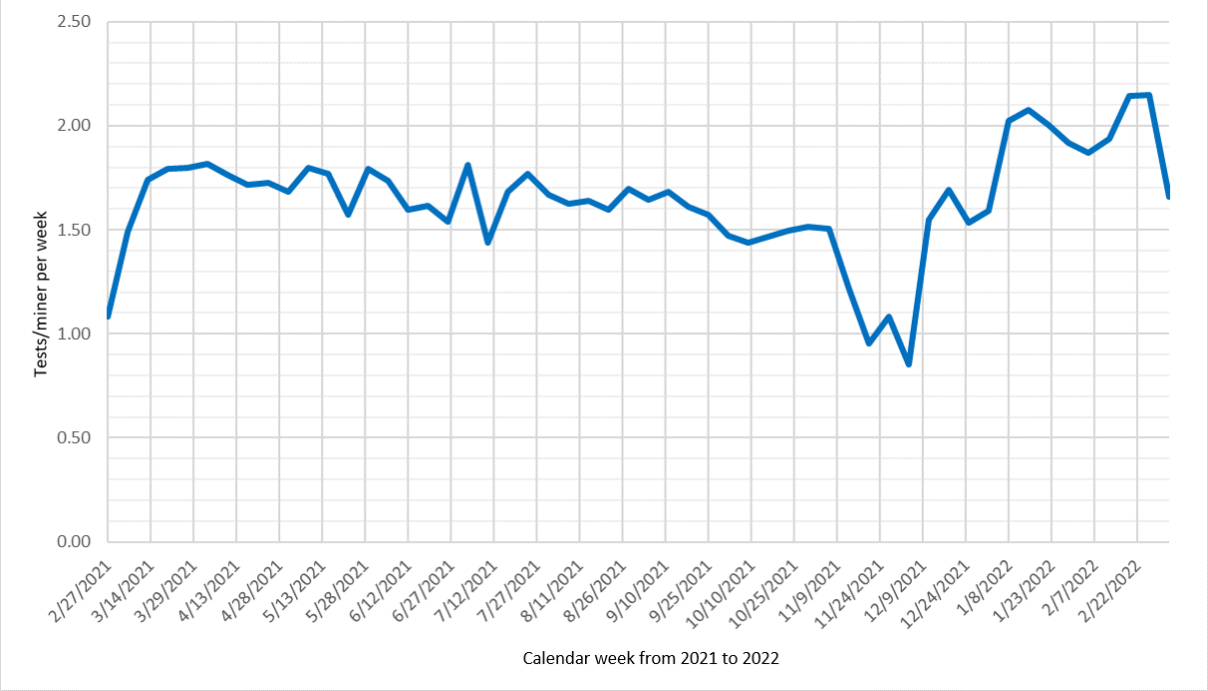
Number of SARS-CoV-2 antigen tests performed per miner per week. Intervention tests were performed at each alternate work shift (schedule 4 days on and 4 days off) during the 2021 calendar weeks 8-52 and 2022 calendar weeks 1-9 (ie, from the end of February 2021, to the end of February 2022; n=169 miners at the intervention site).

**Figure 2.**
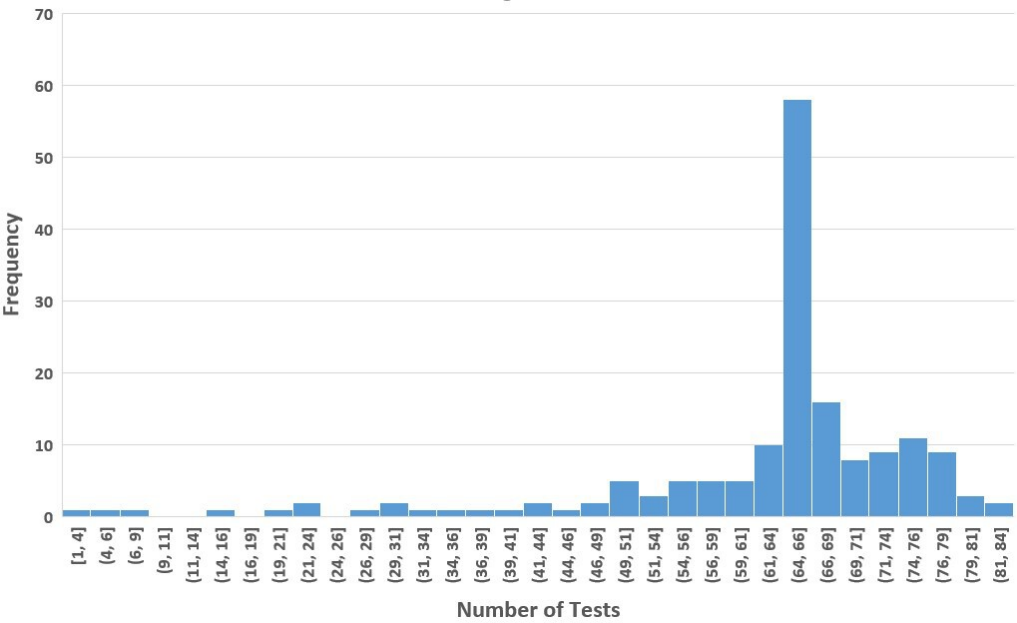
Distribution of the number of SARS-CoV-2 antigen tests per miner. There were approximately 84 opportunities over 267 study days. Participants performed an average of 62.0 (SD 14.4) tests and a median of 65 tests (IQR 68.25-61.75), ranging from 1 to 84 tests (n=169 miners at the intervention site).

The mean intervention test acceptance rate was 96.4% (11,413/11,842). The high test acceptance was unaffected by demographic or medical history characteristics or baseline COVID-19 vaccine uptake (*P*>.05 for all, data not shown). The number of tests per miner per week (mean 1.64, SD 0.26) did not show a trend over the study (Figure 1; slope=0.004; SE=0.005; *P*=.46; temporal autocorrelation=–0.73). The total number of testing opportunities varied by the individual, with an average of 84 opportunities per miner over 267 study days. Participants voluntarily performed an average of 62.0 (SD 14.4) tests, ranging from 1 to 84 tests (Figure 2).

### Diagnostic Accuracy of the Intervention Test Compared to RT-PCR Gold Standard Testing

Sensitivity, specificity, and predictive values of the intervention (QAT on self-collected NSs at the mine site analyzed by trained miner investigators in the temporary field laboratory) were established through comparison with the gold standard test (ie, qualitative RT-PCR conducted on an NPS collected on the same day by trained health personnel and analyzed in a laboratory). Compared to gold standard testing on a sample of 68 pairs of tests, the intervention test in the field setting showed 100% sensitivity and 100% specificity (Tables2  3).

**Table 2.** Comparison of test characteristics between the qualitative rapid antigen test on midnasal swabs in the field and the gold standard test in a laboratory, in a sample of 68 pairs of tests.

Test result	Gold standard test (qualitative RT-PCR^a^ on NPSs^b^)		
	Disease (positive)	Nondisease (negative)	Total
Antigen test on NS^c^			
Positive	38	0	38
Negative	0	30	30
Total	38	30	68

a RT-PCR:reverse transcriptase polymerase chain reaction.

b NPS: nasopharyngeal swab.

c NS: nasal swab.

**Table 3.** Sensitivity, specificity, and predictive values for the qualitative rapid antigen test on midnasal swabs in the field, compared to the gold standard test in a laboratory, in a sample of 68 pairs of tests.

Parameters	Equations	Calculated sensitivity (95% CI)
Sensitivity (%)	True positive/(true positive + false negative)	100 (91-100)
Specificity (%)	True negative/(true negative + false positive)	100 (88-100)
Positive predictive value (%)	True positive/(true positive + false positive)	100^a^
Negative predictive value (%)	true negative/(true negative + false negative)	100^a^

a 95% CI was not calculated for these values.

### Diagnostic Accuracy of Self-Collected NS Versus NPS Collected by Health Personnel Using RT-qPCR

For this analysis, 46 paired samples (NS and NPS) were collected. Among these swabs, the human RNP gene was not detected in 4 samples: 3 from self-collected samples and 1 NPS collected from a health care professional following RT-qPCR conducted in the Center for Global Health Perkins laboratories. These samples were excluded from further analyses. The RT-qPCR results for the 42 remaining samples are shown in Table 4.

**Table 4.** Diagnostic performance of nasal swab versus nasopharyngeal swab using RT-qPCR^a^ for SARS-CoV-2 (42 matched samples). Samples with a cycle threshold value of ≥40 were considered negative.

Test result	Nasopharyngeal swab		Concordance rate (%)	Kappa coefficient (95% CI)
	Negative	Positive		
Nasal swab			80.9	0.39 (0.04‐0.73)
Negative	4	5		
Positive	3	30		

a RT-qPCR: quantitative reverse transcriptase polymerase chain reaction.

**Figure 3.**
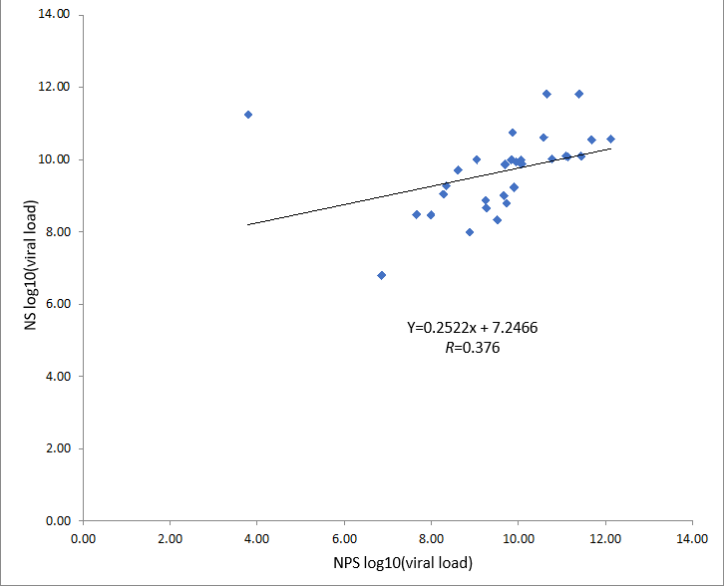
Modest correlation between SARS-CoV-2 viral load from nasopharyngeal swabs and nasal swabs. A linear regression model was performed from nasopharyngeal swab and nasal swab paired samples SARS-CoV-2 viral load (log10) to examine the relationship between viral loads and the two sample collection methodologies (n=30; y=0.2522x+7.2466; *R*=0.376). NPS: nasopharyngeal swab; NS: nasal swab.

The concordance rate was 80.9% between the two sampling methods with a κ coefficient of 0.39 (95% CI 0.04‐0.73; *P*=.01). Simple linear regression was performed to further investigate the relationship between viral loads and the two sample collection methodologies (Figure 3). A modest correlation (*R*=0.38; *P*=.04) for SARS-CoV-2 viral load was detected between 30 paired NP and NS samples. The Ct values and computed viral loads for the discordant samples are shown in Table 5. The NPS virus nucleoprotein gene N1 Ct values for the discordant samples were ≥35, which, after normalizing to the respective RNP sample [18], corresponded to viral loads between log 3 to log 6.5 copies of viral RNA per 1000 cells. This magnitude of viral load was not detectable in self-collected NS samples, suggesting that an NPS was better than an NS for detecting lower viral loads.

**Table 5.** Cycle threshold values and corresponding calculated viral loads of discordant self-collected nasal swab and nasopharyngeal samples collected by trained health care providers using RT-qPCR^a^.

Sample ID	NS^b^		NPS^c^		Corresponding viral load (log_10_)	
	N1 Ct^d^	RP^e^ Ct	N1^f^ Ct	RP Ct	NS	NP
221	>40	37.17	36.82	36.59	0^g^	7.31
263	>40	38.27	37.14	33.96	0	6.17
398	>40	30.85	38.43	25.07	0	3.09
420	>40	34.15	37.07	37.47	0	6.26
738	>40	35.64	37.14	33.01	0	5.78
749	38.52	31.72	>40	33.27	5.74	0
759	35.95	26.96	>40	24.58	4.42	0
967	28.59	31.39	>40	32.15	8.43	0

a RT-PCR: reverse transcriptase polymerase chain reaction.

b NS: nasal swab.

c NPS: nasopharyngeal swab.

d Ct: cycle threshold.

e RP: human RNase P gene assay control.

f N1: virus nucleoprotein gene N1 assay.

g Cycle threshold values ≥40 were considered negative.

### Seropositivity Testing

**Figure 4.**
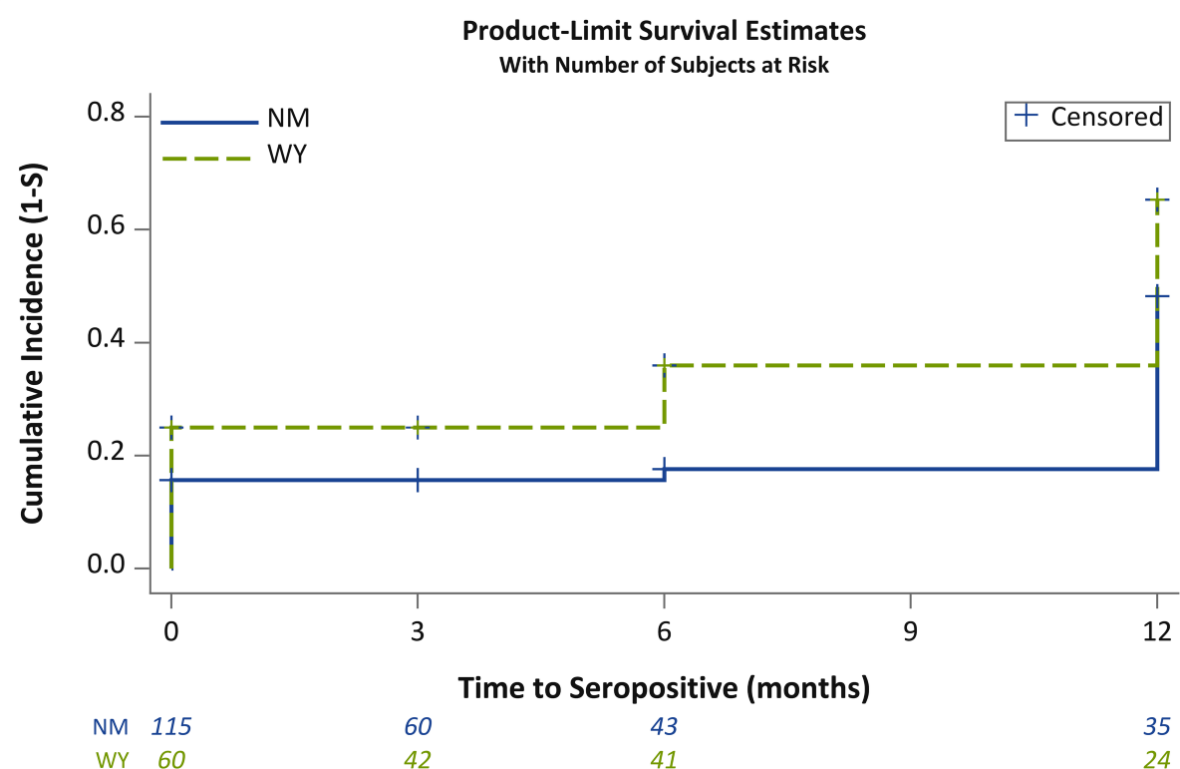
Cumulative incidence of SARS-CoV-2 infection in miners in intervention and control mine sites. Data shown are the cumulative incidences of seropositivity (1 – survival estimated by Kaplan-Meier analysis) for New Mexico–based miners from the intervention mine (solid blue line) and Wyoming-based miners from the control mine (dashed green line). Plus symbols indicate miners that were not followed in subsequent testing periods or were negative at the end of follow-up. Numbers at the bottom of the graph are the number of miners in the analysis at each time. NM: New Mexico; S: survival; WY: Wyoming.

Seropositivity patterns of miners enrolled and tested during the initial recruitment period (baseline, February to March 2021) were compared between intervention and control miners using *χ*^2 ^tests for prevalent seropositivity and cumulative postbaseline incidence during the study. At baseline, prevalent seropositivity was seen among 18 out of 115 (15.7%) intervention miners and 15 out of 60 (25%) control miners (OR 0.56, 95% CI 0.26-1.19; *P*=.13), and cumulative postbaseline incidence was found in 14 out of 97 (14%) intervention miners compared to 17 out of 45 (38%) control miners (OR 0.28, 95% CI 0.12-0.63; *P*=.002; Table 6). We also used Kaplan–Meier analysis and log-rank tests, which accounted for dropouts, to visualize and analyze whether seropositivity patterns were different for intervention and control miners. Figure 4 shows that seropositivity was greater in control miners than in intervention miners at all time periods (log-rank test *P*=.02). The 12-month cumulative incidence was 48.2% (95% CI 35.4-62.9) among intervention miners and 65.3% (95% CI 51.1-79.2) among control miners when estimated by Kaplan-Meier analysis. When only postbaseline incidence was analyzed, the log-rank test had a lower power and was not significant (*P*=.09) with a 12-month seropositivity of the intervention group of 38.6% (95% CI 24.9-56.4) and 53.8% (95% CI 37.2-72.1) for the control group.

**Table 6.** Prevalent and incident seropositivity patterns between the intervention and control miners enrolled and tested during the initial recruitment period (baseline, February to March 2021).

Study timepoint	Intervention (New Mexico–based) mine		Control (Wyoming-based) mine (n=35)	
	New seropositivity, n	Cumulative seropositivity, n/N (%)	New seropositivity, n	Cumulative seropositivity, n/N (%)
Prevalent seropositivity (baseline)	18	18/115 (15.7)	15	15/60 (25)
Postbaseline incident seropositivity^a^				
3 mo	0	0/97 (0)	0	0/45 (0)
6 mo	1	1/97 (1)	6	6/45 (13)
12 mo	13	14/97 (14)	11	17/45 (38)

a Postbaseline incident seropositivity was calculated using the total sample size minus the baseline sample size.

Table S1 in Multimedia Appendix 1 shows unadjusted logistic regression analyses for prevalent and postbaseline incidence, as well as the combined cumulative seropositivity. Combined cumulative seropositivity analyses included miners enrolled at any time during the study. When testing differences in the incidence of seropositivity after baseline, intervention miners (14/97, 14%) had a lower incidence compared to control miners (17/45, 38%; OR 0.28, 95% CI 0.12-0.64; *P*=.002). Incident seropositivity was lower in those who self-reported using masks very often (6/67, 9%) or often (11/39, 28%) compared to those using masks less often (13/35, 37%; *P*=.004; Table S1 in Multimedia Appendix 1). A previous positive COVID-19 test was associated with higher odds of baseline seropositivity (OR 12.72, 95% CI 5.00-32.31; *P*<.001) but not for incident seropositivity (OR 1.34, 95% CI 0.45-4.15; *P*=.59). Age, race and ethnicity, sex, ride-sharing, and COVID-19 vaccination status were not associated with baseline or incident seropositivity. Seropositivity at any time during the study (combined cumulative seropositivity) was lower among intervention miners (OR 0.23, 95% CI 0.12-0.43; *P*<.001), lower among miners who self-reported wearing masks very often (OR 0.19, 95% CI 0.09-0.38; *P*<.001), and lower among miners vaccinated at baseline (OR 0.41, 95% CI 0.19-0.89; *P*=.03). Combined cumulative seropositivity was lower among miners with any nasal testing, which was confounded by the intervention and baseline seropositivity, and odds of cumulative seropositivity did not show a dose-response relationship, with more tests having a lower OR than fewer tests (*P*>.05, data not shown). We also used multivariable logistic models with covariates for age, race and ethnicity, sex, masking frequency, ride-sharing, and COVID-19 vaccination status to assess factors associated with seropositivity (Table S2 in Multimedia Appendix 1). More frequent masking was significantly associated with lower odds of seropositivity for incident and combined cumulative seropositivity measures. Further, more frequent masking was more common in the intervention group, had a strong protective effect on seroprevalence, and confounded the protective effect of the intervention on incident seropositivity and combined cumulative seropositivity.

## Discussion

### Principal Results

Workers in essential sectors have experienced higher COVID-19 infection and mortality rates throughout the pandemic [19]. Much attention has been given to the significant occupational risk of infection among health care workers, but essential work extends beyond health care. Understanding the feasibility and effectiveness of implementing point-of-care testing in essential non–health care professions is crucial for preventing or slowing the transmission of infectious diseases.

Miners, deemed essential workers, face unique challenges that make them more vulnerable and susceptible to COVID-19 [20]. Mining environments often have decreased ventilation and limited availability for physical distancing, remote work, or flexible schedules. Miners are also exposed to higher levels of air pollutant particulates, which may increase their susceptibility to respiratory infections or worsen health outcomes from respiratory infections [21]. Moreover, mine sites are situated in rural areas with limited access to health care and testing facilities. Despite these disproportionate challenges, limited guidelines are available for maintaining mining and other non–health care operations effectively and safely during an infectious disease outbreak. Thus, the objective of this study was to investigate the feasibility and effectiveness of using frequent point-of-care molecular workplace surveillance as an intervention strategy to prevent the spread of SARS-CoV-2 at rural mining sites.

The intervention had a high mean acceptance rate of 96.4% (11,413/11,842), reflecting the involvement of mine safety personnel in the design and execution of the study. Additionally, frequent point-of-care workplace surveillance was associated with a lower cumulative SARS-CoV-2 seropositivity rate in intervention miners compared to control miners (*P*=.002). This study further examined the reliability of self-collected NS versus NPS samples collected by trained health care personnel to detect SARS-CoV-2 using RT-qPCR. The Cohen κ coefficient of 0.39 indicates fair agreement between the two collection methods. However, we observed that NPS samples that tested positive for SARS-CoV-2 by RT-qPCR but had lower viral loads were frequently associated with negative results when tested using NSs instead. While less reliable when compared to NPSs for detecting low levels of SARS-CoV-2, self-collected NS can provide diagnostic performance comparable to NPSs in participants with higher viral loads. As such, NS is a suitable and practical option for intervention test use, especially in resource-limited settings where point-of-care QAT is more accessible, cost-effective, and faster than the gold standard test.

### Limitations

A limitation of this study is that protective behaviors influence COVID-19 health outcomes, including confounding seropositivity rates [22]. While the CDC has encouraged the public use of face coverings during high spikes in community infection rates and vaccination against COVID-19, the use of these protective measures can vary between mine sites based on individual and community factors [23]. We observed that intervention miners were more likely to wear face coverings in public and more willing to receive a vaccine against COVID-19. This difference may reflect partisan variation in official messaging related to COVID-19 protective behaviors to the public between Democratic-governed (New Mexico) and Republican-governed (Wyoming) states [24].

It is also important to note that our study did not involve randomization, so the unmatched distribution of covariates between the two cohorts may result in confounding bias which may have influenced our findings. However, several differences related to demographics and social determinants of health between the mine sites placed the intervention cohort at a higher risk for SARS-CoV-2 infection than the control miners. For example, intervention miners were more likely to be male, which is associated with an increased risk for SARS-CoV-2 infection [25]. Additionally, intervention miners were more likely to be racial and ethnic minorities. Given the disparities observed in minority populations during the pandemic [26,27], this selection bias may have placed the intervention miners at an increased inherent risk for infection and associated complications, which may have biased the protective effect of the intervention toward the null value. Intervention miners were more likely to ride-share and report previous exposure to woodsmoke, a risk factor for increased respiratory disease susceptibility [28]. Moreover, data previously published on our New Mexico cohort indicate that minority miners are at greater risk for developing lung disease than non-Hispanic White miners [29].

Intervention miners also had a lower mean income and educational status than control miners. A study analyzing the joint effects of socioeconomic position, race and ethnicity, and sex on mortality in the first year of the COVID-19 pandemic revealed that COVID-19 mortality was 5 times higher in individuals in low versus high socioeconomic positions [30]. The above-mentioned biases may impact the study’s conclusions, making it difficult to determine whether the observed effects are genuinely due to the intervention or other factors. Although randomization is a key method to mitigate these biases and enhance the credibility of clinical research, community investigators did not consider this study design feasible during the pandemic. Challenges encountered by the study included the lack of easily available intervention tests and community research personnel in the field during the early stage of the pandemic, which limited study recruitment and expansion to other mine sites. The recruitment of a sample size that was lower than planned limited the power of the study. Additionally, the remote location of the study sites increased study costs, and the lack of cellphone and internet connectivity required adaptive strategies by the investigators. Although our intervention can be replicated in other geographic areas and workers in other public and private essential service settings, scaling the intervention would require careful consideration of these unique challenges and a study of its cost-effectiveness.

### Comparison With Prior Work

Since the onset of the pandemic, several papers have been published supporting routine testing to limit the spread of SARS-CoV-2 in health care settings [31-33]. However, studies evaluating frequent point-of-care surveillance, particularly in sectors outside of health care, are still limited. One study, which included a variety of essential workers, including health care workers, found that regular testing of all key workers was associated with reduced transmission of approximately 67 individuals per 1000 tests, with high accuracy of testing (87.1%‐99.9%) [34]. In this study, researchers used RT-PCR rather than QAT, likely due to the limited availability of these tests at the study’s onset. However, a later study evaluating the sensitivity and specificity of the Boson Rapid SARS-CoV-2 rapid antigen detection test revealed an overall sensitivity of 63.04% for anterior NSs and 73.33% for NPSs, with 100% test specificity [35]. A similar study, also showing 100% specificity, compared the Medomics SARS-CoV-2 antigen test device to gold standard testing and reported that rapid antigen detection tests using self-collected anterior NSs proved to be as sensitive as and more tolerable than professionally collected NPSs for Ct values up to 30, determined by RT-PCR [36]. Supporting these results and our own, additional studies have emerged revealing that NS self-sampling yields comparable results to NPS sampling using both RT-PCR [37,38] and QAT for analysis [39]. Studies have established the effectiveness of other field interventions, such as PCR-based wastewater surveillance of SARS-CoV-2 [40]. However, such samples are difficult to collect and analyze in rural areas and provide community-level and not individual-level surveillance data, limiting their impact.

### Conclusion

Our data firmly establish SARS-CoV-2 QAT on self-collected NSs as a feasible alternative to laboratory-based RT-PCR on NPSs for preventing the spread of COVID-19 at essential workplaces within vulnerable communities at an increased risk of being adversely affected by the COVID-19 pandemic because of their occupation, demographics, and rural and remote locations. Additionally, our findings demonstrate the excellent performance of the intervention test in a real-world setting compared to the gold standard. The study findings support developing and implementing policy measures for workplace surveillance and other protective interventions against the spread of SARS-CoV-2 among rural non–health care essential workers. The implications of our findings extend beyond this study, providing valuable insights for designing interventions to maintain employees’ safety at other essential workplaces during an infectious disease outbreak of respiratory pathogens.

## Supplementary material

10.2196/59845 Multimedia Appendix 1 Univariate and multivariable analysis of the association between prevalence seropositivity, postbaseline incident seropositivity, combined cumulative seropositivity, and miner characteristics at baseline.

## Abbreviations

CDC: Centers for Disease Control and Prevention
Ct: cycle threshold
NPS: nasopharyngeal swab
NS: nasal swab
OR: odds ratio
QAT: qualitative SARS-CoV-2 rapid antigen testing
RNP: RNAse P
RT-PCR: reverse transcriptase polymerase chain reaction
RT-qPCR: quantitative reverse transcriptase polymerase chain reaction
