# Supplementary material for: Effectiveness of Frequent Point-of-Care Molecular COVID-19 Surveillance in a Rural Workplace: Nonrandomized Controlled Clinical Trial Among Miners
Source: JMIR Public Health Surveill. 2025 Jan 27;11:e59845. doi: 10.2196/59845 (PMC11790240; doi:10.2196/59845)
Supplement: Multimedia Appendix 1 [file publichealth-v11-e59845-s001.docx]

**Effectiveness of Frequent Point-of-Care Molecular COVID-19 Surveillance in a Rural Workplace: A Non-Randomized Controlled Clinical Trial** **Among Miners**

**Appendix**

**Table S1.** Univariate analysis of the association between prevalence seropositivity, post-baseline incident seropositivity and combined cumulative seropositivity and miner characteristics at baseline. Unadjusted odds ratios (OR) and 95% confidence intervals from logistic regression. Sample sizes for prevalent, post-baseline incident and combined cumulative analyses are different due to analysis inclusion factors. Prevalence seropositivity was calculated based on miners enrolled and first tested at baseline between 23 February and 06 March 2021. Post-baseline incident seropositivity was calculated after removing prevalent miners from the baseline cohort. Combined cumulative seropositivity results are calculated for all miners enrolled and tested, including miners not only from baseline but also some miners enrolled and tested from 3-months, 6-months or 12-months.

|  | **Prevalence Seropositivity** | | | | | **Post-baseline Incident Seropositivity** | | | | | **Combined Cumulative Seropositivity** | | | | |
| --- | --- | --- | --- | --- | --- | --- | --- | --- | --- | --- | --- | --- | --- | --- | --- |
|  | **N** | **N positive** | **% positive** | **OR (95% CI)** | ***P*-value** | **N** | **N positive** | **% positive** | **OR (95% CI)** | ***P*-value** | **N** | **N positive** | **% positive** | **OR (95% CI)** | ***P*-value** |
| **Intervention** | | | | | | | | | | | | | | | |
| Yes (NM) | 115 | 18 | 15.65 | 0.56 (0.26, 1.20) | 0.14 | 97 | 14 | 14.43 | 0.28 (0.12, 0.64) | **0.002** | 169 | 36 | 21.30 | 0.23 (0.12, 0.43) | **<.001** |
| No (WY) | 60 | 15 | 25.00 | reference |  | 45 | 17 | 37.78 | reference |  | 61 | 33 | 54.10 | reference |  |
| **Age, years** | | | | | | | | | | | | | | | |
| Under 40 | 52 | 10 | 19.23 | reference |  | 42 | 9 | 21.43 | reference |  | 80 | 22 | 27.50 | reference |  |
| 40-59 | 94 | 18 | 19.15 | 1.00 (0.42, 2.35) | 0.90 | 76 | 17 | 22.37 | 1.06 (0.42, 2.63) | 0.79 | 113 | 35 | 30.97 | 1.18 (0.63, 2.23) | 0.42 |
| 60-74 | 20 | 3 | 15.00 | 0.74 (0.18, 3.03) |  | 17 | 5 | 29.41 | 1.53 (0.43, 5.48) |  | 24 | 10 | 41.67 | 1.88 (0.73, 4.86) |  |
| **Race/Ethnicity** | | | | | | | | | | | | | | | |
| White | 126 | 22 | 17.46 | reference |  | 104 | 24 | 23.08 | reference |  | 152 | 48 | 31.58 | reference |  |
| American Indian or Alaska Native | 19 | 5 | 26.32 | 1.69 (0.55, 5.17) | 0.57 | 14 | 3 | 21.43 | 0.91 (0.23, 3.53) | 0.97 | 29 | 9 | 31.03 | 0.98 (0.41, 2.30) | 0.99 |
| Some other race | 22 | 5 | 22.73 | 1.39 (0.46, 4.17) |  | 17 | 3 | 17.65 | 0.71 (0.19, 2.70) |  | 34 | 10 | 29.41 | 0.90 (0.40, 2.04) |  |
| Prefer not to answer | 2 | 1 | 50.00 | 4.73 (0.29, 78.50) |  | 1 | 1 | 100.00 | N/A |  | 5 | 2 | 40.00 | 1.44 (0.23, 8.93) |  |
| **Sex** | | | | | | | | | | | | | | | |
| Female | 23 | 2 | 8.70 | reference |  | 21 | 7 | 33.33 | reference |  | 25 | 9 | 36.00 | reference |  |
| Male | 149 | 30 | 20.13 | 2.65 (0.59, 11.92) | 0.66 | 119 | 23 | 19.33 | 0.48 (0.17, 1.32) | 0.36 | 202 | 58 | 28.71 | 0.72 (0.30, 1.71) | 0.90 |
| Intersex | 1 | 1 | 100.00 | N/A |  | N/A | N/A | N/A | N/A |  | 1 | 1 | 100.00 | N/A |  |
| Prefer not to answer | 1 | 0 | 0 | N/A |  | 1 | 1 | 100.00 | N/A |  | 1 | 1 | 100.00 | N/A |  |
| **Mask use** | | | | | | | | | | | | | | | |
| Never, rarely, sometimes | 50 | 15 | 30.00 | reference |  | 35 | 13 | 37.14 | reference |  | 62 | 31 | 50.00 | reference |  |
| Often | 46 | 7 | 15.22 | 0.42 (0.15, 1.15) | 0.05 | 39 | 11 | 28.21 | 0.67 (0.25, 1.77) | 0.004 | 53 | 18 | 33.96 | 0.51 (0.24, 1.10) | <.001 |
| Very Often | 77 | 10 | 12.99 | 0.35 (0.14, 0.86) |  | 67 | 6 | 8.96 | 0.17 (0.06, 0.49) |  | 109 | 17 | 15.60 | 0.19 (0.09, 0.38) |  |
| **Ride sharing** | | | | | | | | | | | | | | | |
| Never, rarely, sometimes | 110 | 21 | 19.09 | reference |  | 89 | 21 | 23.60 | reference |  | 142 | 44 | 30.99 | reference |  |
| Often | 19 | 5 | 26.32 | 1.51 (0.49, 4.67) | 0.37 | 14 | 3 | 21.43 | 0.88 (0.23, 3.47) | 0.85 | 27 | 9 | 33.33 | 1.11 (0.46, 2.67) | 0.58 |
| Very often | 42 | 5 | 11.90 | 0.57 (0.20, 1.63) |  | 37 | 7 | 18.92 | 0.76 (0.29, 1.97) |  | 54 | 13 | 24.07 | 0.71 (0.34, 1.45) |  |
| **Vaccinated** | | | | | | | | | | | | | | | |
| Yes | 37 | 4 | 10.81 | 0.46 (0.15, 1.42) | 0.18 | 33 | 5 | 15.15 | 0.56 (0.20, 1.59) | 0.27 | 52 | 9 | 17.31 | 0.41 (0.19, 0.89) | **0.03** |
| No | 135 | 28 | 20.74 | reference |  | 107 | 26 | 24.30 | reference |  | 174 | 59 | 33.91 | reference |  |
| **Previously positive for COVID** | | | | | | | | | | | | | | | |
| No | 112 | 9 | 8.04 | reference |  | 103 | 23 | 22.33 | reference |  | 151 | 35 | 23.18 | reference |  |
| Yes | 38 | 20 | 52.63 | 12.72 (5.00, 32.31) | <.001 | 18 | 5 | 27.78 | 1.34 (0.43, 4.15) | 0.59 | 42 | 26 | 61.90 | 5.39 (2.60, 11.16) | <.001 |
| Never tested | 25 | 4 | 16.00 | 2.18 (0.61, 7.75) |  | 21 | 3 | 14.29 | 0.58 (0.16, 2.14) |  | 37 | 8 | 21.62 | 0.91 (0.38, 2.18) |  |

Note: N/A = not analyzed.

**Table S2.** Multivariable analysis of the association between prevalence seropositivity, post-baseline incident seropositivity and combined cumulative seropositivity and miner characteristics at baseline. Adjusted odds ratios (OR) and 95% confidence intervals from logistic regression. Sample sizes for prevalent, post-baseline incident and combined cumulative analyses are different due to analysis inclusion factors. Prevalence seropositivity was calculated based on miners enrolled and first tested at baseline between 23 February and 06 March 2021. Post-baseline incident seropositivity was calculated after removing prevalent miners from the baseline cohort. Combined cumulative seropositivity results are calculated for all miners enrolled and tested, including miners not only from baseline but also some miners enrolled and tested from 3-months, 6-months or 12-months.

|  | **Prevalence Seropositivity** | | | | **Post-baseline Incident Seropositivity** | | | | **Combined Cumulative Seropositivity** | | | |
| --- | --- | --- | --- | --- | --- | --- | --- | --- | --- | --- | --- | --- |
|  | **OR** | **95% CI** | | ***P*-value** | **OR** | **95% CI** | | ***P*-value** | **OR** | **95% CI** | | ***P*-value** |
|  |  | **LB** | **UB** |  |  | **LB** | **UB** |  |  | **LB** | **UB** |  |
| **Intervention** |  |  |  |  |  |  |  |  |  |  |  |  |
| No (WY) | reference | | | | reference | | | | reference | | | |
| Yes (NM) | 0.75 | 0.23 | 2.52 | 0.65 | 0.32 | 0.09 | 1.14 | 0.08 | 0.31 | 0.13 | 0.76 | 0.01 |
| **Age** |  |  |  |  |  |  |  |  |  |  |  |  |
| age under 40 | reference | | | | reference | | | | reference | | | |
| age 40-59 | 1.20 | 0.44 | 3.29 | 0.94 | 1.43 | 0.45 | 4.55 | 0.21 | 1.59 | 0.73 | 3.45 | 0.07 |
| age 60-74 | 1.06 | 0.22 | 5.13 |  | 5.12 | 0.82 | 31.97 |  | 4.59 | 1.25 | 16.90 |  |
| **Race/Ethnicity** |  |  |  |  |  |  |  |  |  |  |  |  |
| White | reference | | | | reference | | | | reference | | | |
| American Indian or Alaska Native | 2.66 | 0.69 | 10.20 | 0.39 | 1.40 | 0.26 | 7.62 | 0.60 | 2.36 | 0.82 | 6.79 | 0.22 |
| Some other race | 1.69 | 0.44 | 6.46 |  | 3.23 | 0.59 | 17.53 |  | 2.41 | 0.87 | 6.69 |  |
| prefer not to answer | 5.26 | 0.24 | 117.20 |  | N/A | N/A | N/A |  | 5.70 | 0.76 | 42.77 |  |
| **Sex** |  |  |  |  |  |  |  |  |  |  |  |  |
| female | reference | | | | reference | | | | reference | | | |
| male | 3.22 | 0.66 | 15.71 | 0.15 | 0.41 | 0.13 | 1.31 | 0.13 | 0.72 | 0.26 | 1.98 | 0.52 |
| **Mask use** |  |  |  |  |  |  |  |  |  |  |  |  |
| Never, Rarely, Sometimes | reference | | | | reference | | | | reference | | | |
| Often | 0.42 | 0.11 | 1.57 | 0.28 | 1.94 | 0.52 | 7.21 | 0.03 | 1.01 | 0.39 | 2.63 | 0.01 |
| Very Often | 0.38 | 0.11 | 1.33 |  | 0.34 | 0.08 | 1.47 |  | 0.29 | 0.11 | 0.75 |  |
| **Ride sharing** |  |  |  |  |  |  |  |  |  |  |  |  |
| Never, Rarely, Sometimes | reference | | | | reference | | | | reference | | | |
| Often | 1.71 | 0.40 | 7.32 | 0.59 | 2.23 | 0.40 | 12.45 | 0.63 | 2.32 | 0.77 | 6.98 | 0.31 |
| Very Often | 0.74 | 0.22 | 2.51 |  | 1.46 | 0.41 | 5.17 |  | 1.46 | 0.59 | 3.64 |  |
| **Vaccinated** |  |  |  |  |  |  |  |  |  |  |  |  |
| no | reference | | | | reference | | | | reference | | | |
| yes | 0.79 | 0.23 | 2.69 | 0.70 | 0.60 | 0.16 | 2.19 | 0.44 | 0.56 | 0.22 | 1.41 | 0.22 |

Note: The statistical model included all variables shown in the table. Not included were previously positive test for COVID-19. N/A = not analyzed; OR = odds ratio; UB – upper bounds; LB= lower bounds
